# Association of smoking with incident CKD risk in the general population: A community-based cohort study

**DOI:** 10.1371/journal.pone.0238111

**Published:** 2020-08-27

**Authors:** Wonji Jo, Sangmi Lee, Young Su Joo, Ki Heon Nam, Hae-Ryong Yun, Tae Ik Chang, Ea wha Kang, Tae-Hyun Yoo, Seung Hyeok Han, Shin-Wook Kang, Jung Tak Park

**Affiliations:** 1 Department of Internal Medicine, College of Medicine, Institute of Kidney Disease Research, Yonsei University, Seoul, Korea; 2 Division of Nephrology, Department of Internal Medicine, Myongji Hospital, Goyang, Gyeonggi-do, Republic of Korea; 3 Division of Integrated Medicine, Department of Internal Medicine, College of Medicine, Yonsei University, Seoul, Korea; 4 Department of Internal Medicine, National Health Insurance Service Medical Center, Ilsan Hospital, Goyang, Gyeonggi-do, Korea; 5 Department of Internal Medicine, College of Medicine, Severance Biomedical Science Institute, Brain Korea 21 PLUS, Yonsei University, Seoul, Korea; University of Calfornia San Francisco, UNITED STATES

## Abstract

**Background:**

Chronic kidney disease (CKD) is a public health problem, and an unfavorable lifestyle has been suggested as a modifiable risk factor for CKD. Cigarette smoking is closely associated with cardiovascular disease and cancers; however, there is a lack of evidence to prove that smoking is harmful for kidney health. Therefore, we aimed to determine the relationship between cigarette smoking and CKD among healthy middle-aged adults.

**Methods:**

Using the database from the Korean Genome and Epidemiology Study, we analyzed 8,661 participants after excluding those with baseline estimated glomerular filtration rate (eGFR)<60 ml/min/1.72 m^2^ or proteinuria. Exposure of interest was smoking status: never-, former-, and current-smokers. Primary outcome was incident CKD defined as eGFR <60 ml/min/1.73 m^2^ or newly developed proteinuria.

**Results:**

The mean age of the subjects was 52 years, and 47.6% of them were males. There were 551 (6.4%) and 1,255 (14.5%) subjects with diabetes and hypertension, respectively. The mean eGFR was 93.0 ml/min/1.73 m^2^. Among the participants, 5,140 (59.3%), 1,336 (15.4%), and 2,185 (25.2%) were never-smokers, former-smokers, and current-smokers, respectively. During a median follow-up of 11.6 years, incident CKD developed in 1,941 (22.4%) subjects with a crude incidence rate of 25.1 (24.0–26.2) per 1,000 person-years. The multivariable Cox regression analysis after adjustment of confounding factors showed hazard ratios (95% confidence interval) of 1.13 (0.95–1.35) and 1.26 (1.07–1.48) for CKD development in the former- and current-smokers, compared with never-smokers.

**Conclusion:**

This study showed that smoking was associated with a higher risk of incident CKD among healthy middle-aged adults.

## Introduction

Chronic kidney disease (CKD) is a growing public health problem. According to the Global Burden of Disease Study 2016, more than 200 million people worldwide have CKD [1]. In addition, in the United States, the number of patients with end-stage renal disease (ESRD) who require renal replacement therapy (RRT) is more than 400,000 [2]. Many studies identified risk factors, such as advanced age, hypertension, diabetes mellitus (DM), dyslipidemia, obesity, and usage of nephrotoxic agents, for CKD [3–9], Because CKD is accompanied by concomitant disease burden, increased mortality, and high healthcare cost, management of the modifiable risk factors should be the key strategy for prevention of CKD.

Cigarette smoking is a leading cause for preventable death from cardiovascular disease (CVD) and cancer [10]. Although controversial, several studies have shown that the risk of incident CVD decreases with increase in the smoking cessation period [11–14]. In addition, smoking cessation at an earlier age is associated with lower risk of death [15, 16]. Therefore, cigarette smoking is considered an important modifiable risk factor, and smoking cessation has become a major target policy for many countries. Smoking promotes atherosclerosis and vascular dysfunction; hence, it can cause damage to other organs, including the kidney. In fact, many epidemiologic studies to date have demonstrated the negative impacts of smoking on kidney health [17–25]. Although there have been several observational studies that determined the effects of smoking exposure on kidney function, assessments comparing the risk of kidney damage with those who stopped smoking after active smoking periods have been limited. In addition, the duration of most previous reports was relatively short and could not evaluate the long-term effects of smoking on kidney function. [20, 26–28].

In this study, we sought to clarify the long-term relationship between smoking and kidney function in healthy middle-aged Korean adults. In addition, we examined whether smoking cessation can help prevent kidney disease.

## Materials and methods

### Study design and subjects

**Fig 1:**
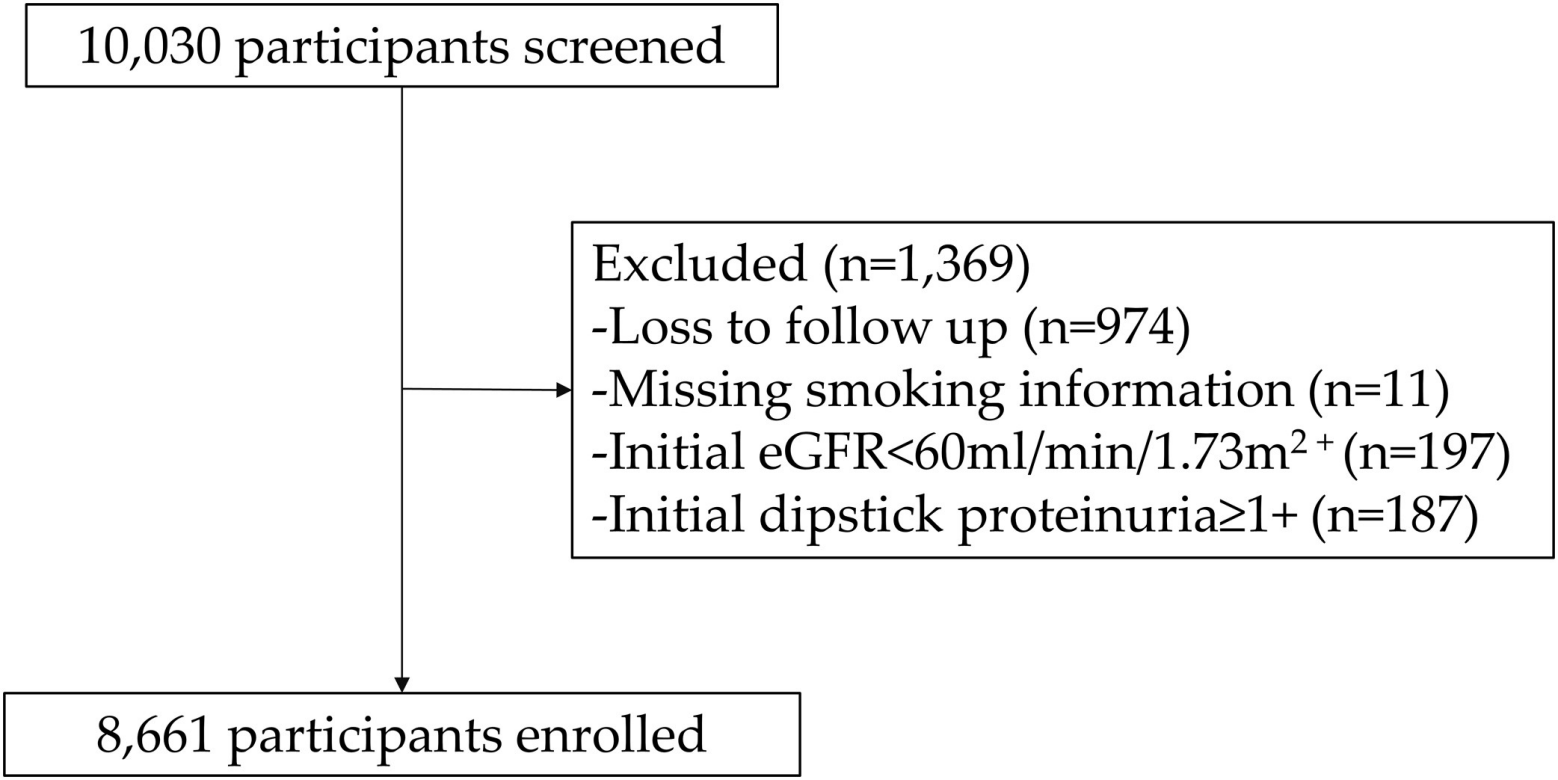
Flow diagram of the study cohort. *Abbreviation*: eGFR, estimated glomerular filtration rate.

The Korean Genome and Epidemiology Study (KoGES) is a population-based cohort study conducted in rural (Ansung) and urban (Ansan) areas of South Korea. This study was designed to elucidate the interaction between lifestyle factors and genetic risk factors in the development of non-communicable diseases, including CKD. The detailed profile and methods on construction of the KoGES cohort are described elsewhere [29]. Briefly, 10,030 participants were enrolled between 2001 and 2002. Biannual health examinations and surveys were provided to the participants until 2014, and the retention rate was 62.8% at the end of the 6^th^ follow-up phase. We excluded (a) patients with estimated glomerular filtrating rate (eGFR) <60 mL/min/1.73 m^2^, (b) those with urine dipstick positive proteinuria ≥ 1+ at baseline, (c) those with missing follow-up data for serum creatinine level or urinary dipstick test, and (d) those without available information for smoking status (Fig 1). Thus, 8,661 participants were included in the final analysis. All participants provided informed consent. The present study was carried out in accordance with the Declaration of Helsinki and was approved by the Institutional Review Board of Yonsei University Health System Clinical Trial Center (4-2016-0100).

### Data measurement

Demographic and socioeconomic data, including age, sex, medical history, education and income levels, smoking status, and alcohol intake were obtained using a standardized self-administered questionnaire at enrollment. Patients with a history of alcohol intake were categorized as non- or current drinkers. Blood pressure was measured by trained nurses using a standard mercury sphygmomanometer with the subject in the sitting position after a 5-min rest. Blood and urine samples were obtained after an 8-h fast and transported to a central laboratory (Seoul Clinical Laboratories, Seoul, Republic of Korea) within 24 hours of sampling. Serum concentrations of blood urea nitrogen, creatinine, albumin, glucose, total cholesterol, triglyceride, high-density lipoprotein cholesterol, low-density lipoprotein cholesterol (LDL-C), and C-reactive protein (CRP) were measured. Urine samples were collected in the morning after the first voiding and were subjected to a dipstick test (URISCAN Pro II; YD Diagnostics Corp., Seoul, Korea). The presence of proteinuria was defined as protein level 1+ in the dipstick urine test. The eGFR was calculated using the CKD epidemiology collaboration equation.

### Exposure and study outcome

The primary exposure of interest was smoking status, and the subjects were classified as non-, former-, and current-smokers. Data for age at initiation of smoking, number of years smoked, and average number of packs of cigarettes smoked per day were also collected from the former- and current-smokers. The smoking pack-years were calculated as the number of years smoked × the average number of packs of cigarettes smoked per day. The surveys collected at enrollment and the second visit were used to assess baseline smoking status.

The primary outcome was incident CKD, which was defined as the occurrence of eGFR <60 ml/min/1.73 m^2^ or newly developed proteinuria (defined as urine dipstick protein≥1+). The study observation ended on December 31, 2014.

### Statistical analysis

All statistical analyses were performed using STATA version 15.1 (StataCorp, College Station, TX, USA). For missing data imputation, the MICE (multivariate imputation by chained equations) method of multiple multivariate imputation in STATA was used. The missing data analysis procedures used missing at random assumptions (S1 Table). We independently analyzed 5 copies of the data, each with missing values suitably imputed, in the multivariable cox regression analyses. The estimates of the variables were averaged to give a single mean estimate and standard errors were adjusted according to Rubin’s rules [30]. The continuous variables were expressed as mean ± standard deviation or median and interquartile ranges, while the categorical variables were expressed as frequencies with percentages. All data were tested for normality before statistical analysis. The Kolmogorov-Smirnov test was performed to determine the normality of distribution of the parameters. Comparisons between groups were performed using analysis of variance with a normal distribution, and the chi-square test or Fisher’s exact test was used for analysis of the categorical variables. Data that did not show a normal distribution were compared using the Kruskal-Wallis test. Cumulative renal survival rates were estimated by Kaplan-Meier analysis and a log-rank test. Multivariable Cox proportional hazard models with a 3-step adjustment level were constructed to determine the independent association of smoking status with incident CKD. The proportionality assumptions for the cause-specific models were tested through inspection of log (-log [survival]) curves, and no violations were observed. Multivariable models were constructed with adjustment for confounding factors. Variables that showed statistical significance in univariable regression analyses were selected for the multivariable model. Model 1 was adjusted for age and sex. Model 2 was further adjusted for comorbidities and socioeconomic factors such as, hypertension, DM, alcohol status, education, income level, and BMI. Model 3 was fully adjusted for the above factors plus laboratory results including albumin and total cholesterol level. The results of the Cox models were presented as HR and 95% confidence interval (CI). Patients who were lost to follow-up or death were censored at the date of the last examination. To test the dose-response relationship between smoking load and incident CKD, we repeated the same Cox analysis after the smokers were classified by 15-year increment in pack-years of smoking. We also performed the subgroup analyses across the following subgroups: age (<60 and ≥60 years), sex, hypertension, DM, body mass index (BMI) (<25 and ≥25 kg/m^2^), alcohol status, and income and education levels. In addition, the association between smoking cessation and risk of the adverse kidney outcome among former-smokers classified by 10-year increments of cessation duration was assessed. For subgroup analysis, the interaction terms between each stratifying factor and smoking status on incident CKD was assessed in adjusted cause-specific proportional hazard models using the likelihood ratio test. Adjustments were made with the fully adjusted model 3 of the multivariable Cox proportional hazard models. Linear mixed-effect models of annual eGFR decline according to smoking status and timed updated smoking status were constructed with adjustment of confounding factors. For all analyses, *p*<0.05 was considered statistically significant.

## Results

### Baseline characteristics

The baseline characteristics by smoking status are shown in Table 1. The mean age of the study cohort was 52 years and 47.6% were men. There were 551 (6.4%) subjects with diabetes and 1,255 (14.5%) with hypertension. The mean eGFR was 93.0 ml/min/1.73 m^2^. Among the participants, 5,140 (59.3%), 1,336 (15.4%), and 2,185 (25.2%) were never-smokers, former-smokers, and current-smokers, respectively. There were more patients with diabetes but fewer patients with hypertension among smokers than among never smokers. Smokers consumed more alcohol and had higher levels of education and income than non-smokers. Baseline kidney function was slightly better in non-smokers than in smokers.

**Table 1:** Baseline characteristics of participants according to smoking status.

Variables	Total	Never smokers	Smokers		
			Ever smokers	Former smokers	Current smokers
	(n = 8,661)	(n = 5,140)	(n = 3,521)	(n = 1,336)	(n = 2,185)
**Smoking**					
Smoking period (years)	10.0±14.1		24.5±11.4	20.1±11.4	27.2±10.5
Smoking load (pack-year)	9.5±15.9		23.3±17.2	21.0±18.6	24.8±16.2
**Age (years)**	52.1±8.8	52.3±8.8	51.7±8.8	52.4±9.0	51.3±8.7
**Male**, **n (%)**	4,124 (47.6)	818 (15.9)	3,306 (93.9)	1,283 (96.0)	2,023 (92.6)
**Body mass index (kg/m**^**2**^**)**	24.6±3.2	24.8±3.2	24.2±3.0	24.5±2.8	23.9±3.1
**Systolic BP (mmHg)**	121.3±18.2	121.3±19.0	121.2±17.1	123.1±17.3	120.1±16.8
**Diastolic BP (mmHg)**	80.2±11.4	79.5±11.6	81.3±10.9	82.5±11.0	80.5±10.7
**Comorbidities**, **n (%)**					
Hypertension	1,255 (14.5)	810 (15.8)	445 (12.6)	217 (16.2)	228 (10.4)
Diabetes	551 (6.4)	294 (5.7)	257 (7.3)	111 (8.3)	146 (6.7)
Cardiovascular disease	63 (0.7)	39 (0.8)	24 (0.7)	15 (1.1)	9 (0.4)
**Alcohol**, **n (%)**	4,096 (47.3)	1,548 (30.1)	2,548 (72.4)	903 (67.6)	1,645 (75.3)
**Education**^b^, **n (%)**					
Low	2,857 (33.0)	2,087 (40.6)	770 (21.9)	264 (19.8)	506 (23.2)
Middle	4,590 (53.0)	2,567 (49.9)	2,023 (57.5)	760 (56.9)	1,263 (57.8)
High	1,214 (14.0)	486 (9.5)	774 (20.7)	312 (23.4)	416 (19.0)
**Income**^c^, **n (%)**					
Low	2,991 (34.5)	1,967 (38.3)	1,024 (29.1)	342 (25.6)	682 (31.2)
Middle	4,036 (46.6)	2,313 (45.0)	1,723 (48.9)	666 (49.9)	1,057 (48.4)
High	1,634 (18.9)	860 (16.7)	774 (22.0)	328 (24.6)	446 (20.4)
**Laboratory findings**					
WBC count (10^3^/μL)	6.5±1.8	6.3±1.7	6.9±1.9	6.4±1.6	7.2±1.9
Hemoglobin (g/dL)	13.6±1.6	12.9±1.4	14.6±1.2	14.6±1.2	14.7±1.2
Albumin (g/dL)	4.2±0.3	4.2±0.3	4.3±0.4	4.4±0.4	4.3±0.3
eGFR (mL/min/1.73m^2^)	93.0±13.0	93.7±12.8	91.9±13.3	90.2±13.4	92.9±13.1
Total cholesterol (mg/dL)	190.6±35.0	190.0±34.4	191.3±35.9	194.0±35.5	189.7±36.1
LDL-cholesterol (mg/dL)	113.7±33.1	114.8±31.5	112.2±35.2	115.5±34.6	110.1±35.4
HDL-cholesterol (mg/dL)	44.7±10.0	45.3±9.9	43.7±10.0	43.9±9.6	43.6±10.2
Triglyceride^a^ (mg/dL)	135 [99, 189]	127 [95, 176]	148 [110, 210]	146 [108, 200]	149 [110, 217]
CRP^a^ (mg/dL)	0.14 [0.07, 0.25]	0.14 [0.07, 0.24]	0.15 [0.07, 0.26]	0.14 [0.07, 0.26]	0.15 [0.07, 0.26]

All data are expressed as mean ± SD or ^a^ median (and interquartile range).

^b^ Low, primary education; middle, secondary education; and high, tertiary education.

^c^ Low, <1,000,000; ≥1,000,000 and <3,000,000; and high, ≥3,000,000 won per month.

***Abbreviations***: BP, blood pressure; WBC, white blood cell; eGFR, estimated glomerular filtration rate; HDL, high-density lipoprotein; LDL, low-density lipoprotein, and CRP, high-sensitivity C-reactive protein.

### Risk of the incident CKD according smoking status

**Fig 2:**
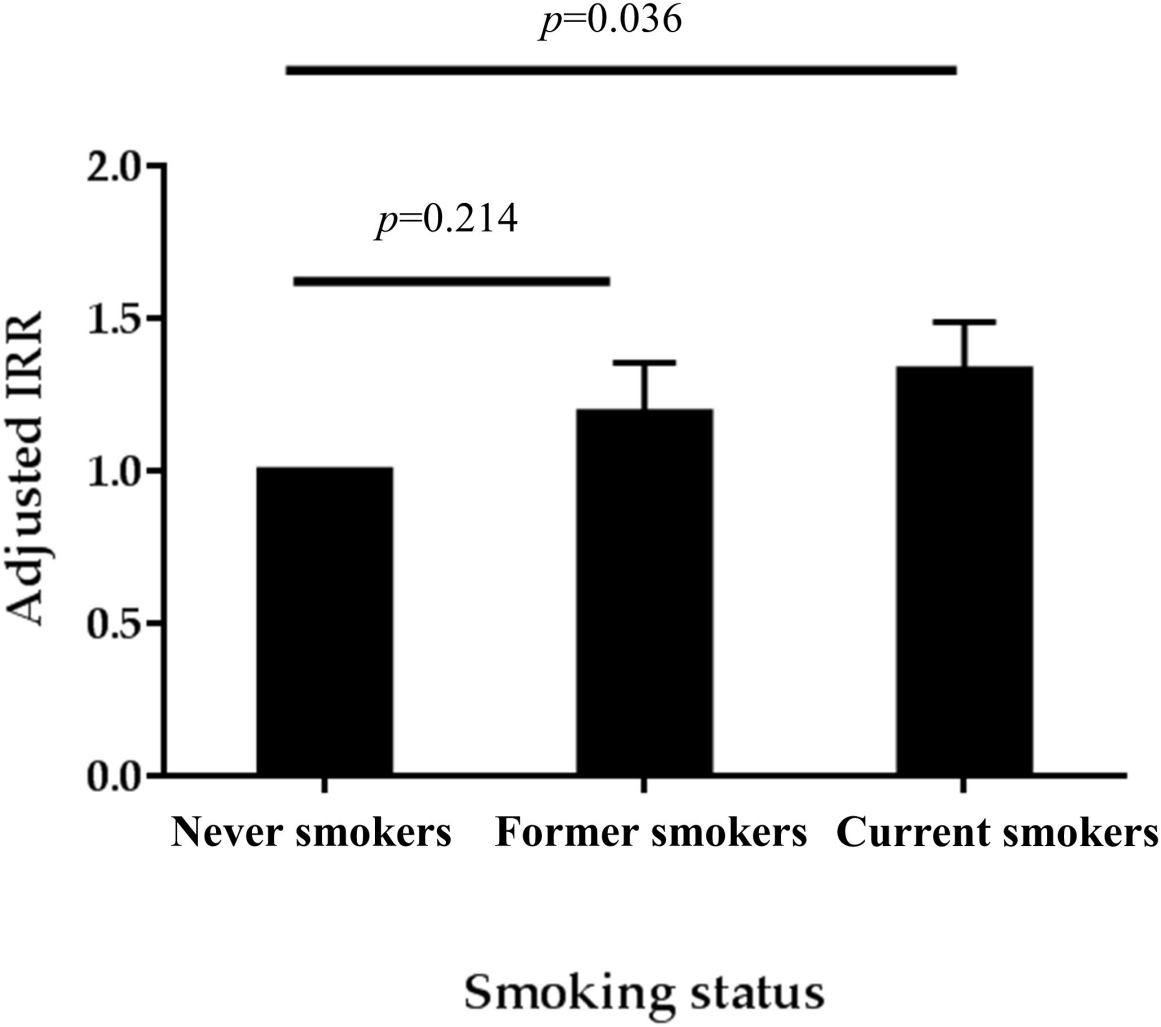
Adjusted incidence rate ratio of CKD according to smoking status. *Abbreviation*: IRR, incidence rate ratio.

**Fig 3:**
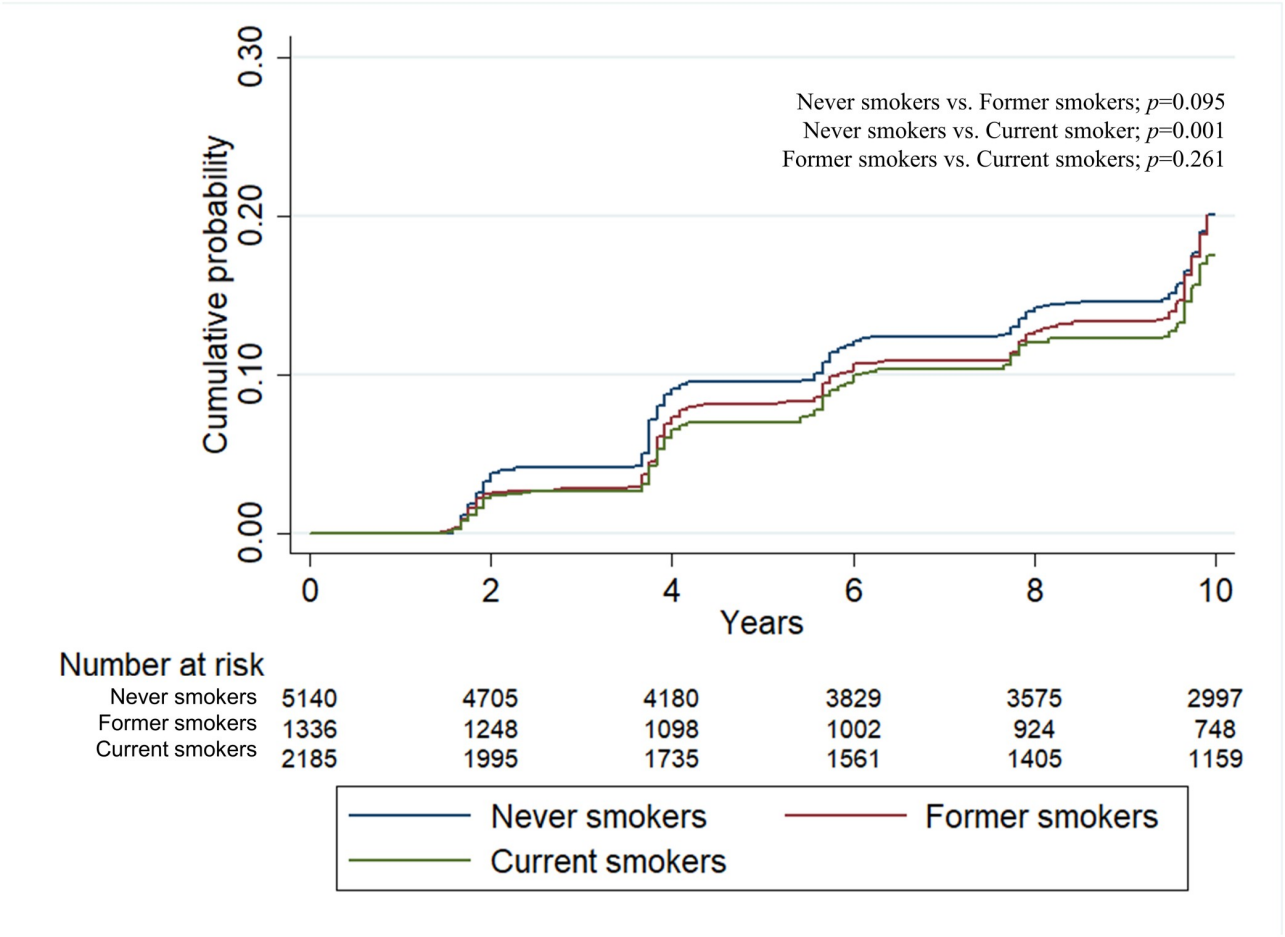
Kaplan-Meier curve for incident CKD.

During a median follow-up of 11.6 years, incident CKD developed in 1,941 (22.4%) subjects with a crude incidence rate of 25.1 (24.0–26.2) per 1,000 person-years. Poisson regression after adjustment of confounding factors showed that the incidence rate ratio (IRR) of incident CKD was highest in the current smoking group (Fig 2). IRRs (95% confidence interval) were 1.11 (0.94–1.33) and 1.19 (1.01–1.39) in the former smokers and current smokers, compared with never smokers. The Kaplan-Meier plots showed that the time to the development of incident CKD was significantly longer in never smokers than current smokers or former smokers (Fig 3). The multivariable Cox regression analysis after adjustment of confounding factors showed hazard ratios (95% CI) of 1.13 (0.95–1.35) and 1.26 (1.07–1.48) for CKD development in the former- and current-smokers, compared with never-smokers (Table 2). Additionally, linear mixed-effects models were constructed, using repeated eGFR measurements of each participant, to evaluate the effect of smoking status on the decline slope of eGFR. The annual eGFR decline was more rapid among current smokers (-1.569 mL/min/1.73m^2^/year) compared to never smokers (-1.282 mL/min/1.73m^2^/year, *p*<0.001) or former smokers (-1.274 mL/min/1.73m^2^/year, *p*<0.001) (S2 Table). A similar relationship between smoking status and annual eGFR decline was also found when smoking status was treated as a time dependent variable (S3 Table).

**Table 2:** Hazard ratios for incident CKD by smoking status.

	Model 1		Model 2		Model 3	
	HRs (95% CI)	*p*	HRs (95% CI)	*p*	HRs (95% CI)	*p*
**Never smokers**	Reference		Reference		Reference	
**Former smokers**	1.14 (0.95–1.35)	0.140	1.13 (0.95–1.35)	0.174	1.13 (0.95–1.35)	0.164
**Current smokers**	1.15 (0.99–1.35)	0.075	1.25 (1.07–1.47)	0.006	1.26 (1.07–1.48)	0.005

**Model 1**: Adjusted for age and sex.

**Model 2**: Model 1 + HTN, DM, alcohol, education, income, and BMI.

**Model 3**: Model 2 + Albumin and total cholesterol.

***Abbreviations***: HR, hazard ratio; CI, confidence interval; eGFR, estimated glomerular filtration; HTN, hypertension; DM, diabetes mellitus; BMI, body mass index.

### Dose-dependent relationship between smoking load and incident CKD

Next, we analyzed the association between smoking load and incident CKD. Smokers were classified into 3 groups by pack-years of smoking (15-year increments). The HRs (95% CIs) were 1.14 (0.94–1.37), 1.07 (0.85–1.34), and 1.12 (0.93–1.36) for- <15, 15–29, and ≥30 pack-years of smoking, respectively, as compared to never-smokers (Table 3). However, the differences were not statistically significant.

**Table 3:** Hazard ratios for incident CKD according to smoking load.

	Model 1		Model 2		Model 3	
	HRs (95% CI)	*p*	HRs (95% CI)	*p*	HRs (95% CI)	*p*
**Never smokers**	Reference		Reference		Reference	
**Smoking load**						
0–15 pack x year	1.11 (0.94–1.32)	0.222	1.16 (0.97–1.38)	0.101	1.14 (0.94–1.37)	0.187
15–30 pack x year	1.04 (0.85–1.28)	0.683	1.12 (0.91–1.38)	0.277	1.07 (0.85–1.34)	0.567
>30 pack x year	1.14 (0.95–1.36)	0.150	1.19 (1.00–1.42)	0.054	1.12 (0.93–1.36)	0.229

**Model 1**: Adjusted for age and sex.

**Model 2**: Model 1 + HTN, DM, alcohol, education, income, and BMI.

**Model 3**: Model 2 + Albumin and total cholesterol.

***Abbreviations***: HR, hazard ratio; CI, confidence interval; eGFR, estimated glomerular filtration; HTN, hypertension; DM, diabetes mellitus; BMI, body mass index.

### Subgroup analysis

Further examinations were done to evaluate the association between smoking and kidney outcome in subgroups stratified by sex, age (<60 or ≥60 years), presence of hypertension and diabetes, BMI (<25 or ≥25 kg/m^2^), alcohol status, and income and educational levels. When the interaction terms between each stratifying factor and the effect of current-smoking on incident CKD was assessed, no significant interactions were found in any of the subgroups. This suggests that the increased incident CKD risk in current smokers compared to never smokers were consistently significant across these subgroups (S4 Table).

### Smoking cessation and risk of incident CKD

Finally, to assess whether smoking cessation can aid in preventing CKD development, we subdivided the former smokers into three groups by cessation duration in 10-year increments. After adjusting for the confounding factors, smoking cessation for 20 years and more showed relationship with lower risk of incident CKD (HR, 0.76; 95% CI, 0.62–0.94), while quitting smoking for less than 20 years was not (Table 4).

**Table 4:** Hazard ratios for incident CKD according to duration of smoking cessation.

	Model 1		Model 2		Model 3	
	HRs (95% CI)	*p*	HRs (95% CI)	*p*	HRs (95% CI)	*p*
**Current smokers**	Reference		Reference		Reference	
**Cessation duration**						
0–10 years	0.81 (0.61–1.07)	0.145	0.77 (0.58–1.01)	0.063	0.83 (0.63–1.09)	0.181
10–20 years	0.88 (0.68–1.16)	0.369	0.84 (0.64–1.10)	0.194	0.89 (0.68–1.17)	0.414
>20 years	0.78 (0.63–0.96)	0.021	0.77 (0.62–0.95)	0.014	0.76 (0.62–0.94)	0.012

**Model 1**: Adjusted for age and sex.

**Model 2**: Model 1 + HTN, DM, alcohol, education, income, and BMI.

**Model 3**: Model 2 + Albumin and total cholesterol.

***Abbreviations***: HR, hazard ratio; CI, confidence interval; eGFR, estimated glomerular filtration; HTN, hypertension; DM, diabetes mellitus; BMI, body mass index.

## Discussion

In this population-based cohort study with a median follow-up of 11.6 years, we found that current smoking was associated with a higher risk of CKD development in middle-aged people without kidney disease. In addition, among former smokers, smoking cessation for ≥ 20 years was associated with a lower risk of incident CKD, suggesting that quitting smoking may be helpful in preventing the development of CKD.

Our findings add evidence to the prevailing notion that smoking is harmful for vascular health. Regarding kidney disease, previous studies have shown that smoking is associated with the presence of proteinuria and impaired kidney function [21–23, 26, 27, 31, 32]. However, our study was different in several aspects. Many previous studies were cross-sectional or retrospective observations [21, 23, 31, 32], did not have follow-up measures for kidney function [22], and defined the study outcome as ESRD [26, 27]. The strengths of the present study lie in the analyses with well-organized longitudinal data from a prospective cohort, long-term observation, and rigorous adjustment of many confounding factors. Because people in our cohort had normal kidney function and few comorbid conditions at study entry, more severe kidney outcomes such as ESRD rarely occurred during follow-up. However, our longitudinal long-term observation over a 11-year duration enabled us to detect early CKD development in healthy middle-aged people without kidney disease. Thus, our findings, along with those of previous studies, confirm the harmful impact of smoking on kidney health in people who do not have overt kidney disease.

To date, few studies have addressed whether smoking is a modifiable factor for the prevention of kidney disease. However, this can be inferred from previous studies in the field of cardiovascular disease research. In a collective analysis using the Cochrane database, quitting smoking was associated with 36% and 32% lower risk of mortality and non-fatal myocardial infarctions, respectively, among patients with coronary heart disease [33]. Accordingly, all guidelines recommend quitting smoking as a first-line measure for the prevention of cardiovascular disease based on many epidemiologic studies [11, 34–36]. The effect of smoking cessation on kidney failure has recently been studied in two large cohorts [26, 37]. Both studies conducted using the Singapore Chinese and Norwegian cohorts showed that the risk of kidney failure, defined as progression to CKD stage 5 or the initiation of dialysis treatment, was lower in people with prolonged smoking cessation. In agreement with these studies, we showed that early smoking cessation was associated with a lower risk of CKD development among former smokers. However, the time elapsed to observe the beneficial effects of smoking cessation appears to be longer because the statistical significance was observed in those who had quit smoking for ≥ 20 years. Presumably, the adverse effects of smoking on the kidney should be taken into account in light of other cardiovascular system disorders, and its cumulative residual effects may last longer than expected even after quitting smoking. Taken together, our findings are informative for establishing a preventive strategy against the development of kidney disease from the viewpoint of public health.

Our study has several limitations. First, this observational study cannot entirely eliminate confounding, although we rigorously adjusted potential factors that might affect clinical outcomes. In addition, causality between smoking exposure and incident CKD is uncertain. However, conducting randomized controlled trials on this issue appear to be infeasible for ethical reasons. Therefore, collecting more evidence regarding the harmful effects of smoking on kidney disease with well-constructed epidemiologic data would be meaningful. Second, information on smoking status relied on a self-reported questionnaire, which might result in inaccurate classification of smokers. Third, in contrast to other western countries [38, 39], only 6.1% of the smokers were women, which is consistent with the Korean national health and nutrition examination survey [40]. The unequal distribution of smokers between men and women may be similar in other Asian countries as reported in different cohort studies [26, 32]. This smaller proportion of women smokers did not allow us to analyze whether smoking effects differ between men and women. Fourth, in this study, the magnitude of HR for CKD development was relatively lower than that in another European cohort study [27], but similar to that in a Singapore-Chinese cohort study [26]. It is unknown whether there is an ethnic difference in kidney injury due to smoking exposure. It should be noted that our cohort comprised healthy adults with few comorbidities. Thus, a 31% higher risk for CKD development in this group may be substantial. In addition, the confidence interval was relatively narrow, suggesting the higher accuracy of this analysis. Finally, the inclusion of only Korean people may limit the generalizability of our findings.

In conclusion, this study showed that cigarette smoking was associated with a higher risk of CKD development in middle-aged healthy adults without kidney disease. Conversely, smoking cessation might be associated with a lower risk of incident CKD among former-smokers. These findings provide robust evidence regarding the harmful effects of smoking on kidney health and highlight the importance of smoking cessation in preventing kidney disease in clinical practice.

## Supporting information

S1 Table Type of missing data in main analysis data. (DOCX)

S2 Table Linear mixed model of annual eGFR decline according to smoking status. (DOCX)

S3 Table Linear mixed model of annual eGFR decline according to time updated smoking status. (DOCX)

S4 Table Interaction terms of stratifying factors and the incident CKD risk of current smokers compared with never smokers. (DOCX)

## References

[1] Global, regional, and national incidence, prevalence, and years lived with disability for 328 diseases and injuries for 195 countries, 1990–2016: a systematic analysis for the Global Burden of Disease Study 2016. Lancet. 2017;390(10100):1211–59. Epub 2017/09/19. 10.1016/S0140-6736(17)32154-2 . 28919117 PMC5605509

[2] Jha V, Garcia-Garcia G, Iseki K, Li Z, Naicker S, Plattner B, et al Chronic kidney disease: global dimension and perspectives. Lancet. 2013;382(9888):260–72. Epub 2013/06/04. 10.1016/S0140-6736(13)60687-X . 23727169

[3] Anderson S, Halter JB, Hazzard WR, Himmelfarb J, Horne FM, Kaysen GA, et al Prediction, progression, and outcomes of chronic kidney disease in older adults. J Am Soc Nephrol. 2009;20(6):1199–209. Epub 2009/05/28. 10.1681/ASN.2008080860 . 19470680

[4] Prakash S, O’Hare AM. Interaction of aging and chronic kidney disease. Semin Nephrol. 2009;29(5):497–503. Epub 2009/09/16. 10.1016/j.semnephrol.2009.06.006 . 19751895 PMC2771919

[5] Shen Y, Cai R, Sun J, Dong X, Huang R, Tian S, et al Diabetes mellitus as a risk factor for incident chronic kidney disease and end-stage renal disease in women compared with men: a systematic review and meta-analysis. Endocrine. 2017;55(1):66–76. Epub 2016/08/02. 10.1007/s12020-016-1014-6 . 27477292

[6] Judd E, Calhoun DA. Management of hypertension in CKD: beyond the guidelines. Adv Chronic Kidney Dis. 2015;22(2):116–22. Epub 2015/02/24. 10.1053/j.ackd.2014.12.001 . 25704348 PMC4445132

[7] Ritz E, Wanner C. Lipid changes and statins in chronic renal insufficiency. Journal of the American Society of Nephrology. 2006;17(SUPPL. 3):S226–S30. 10.1681/ASN.2006080919 17130266

[8] Panwar B, Hanks LJ, Tanner RM, Muntner P, Kramer H, McClellan WM, et al Obesity, metabolic health, and the risk of end-stage renal disease. Kidney Int. 2015;87(6):1216–22. Epub 2014/12/18. 10.1038/ki.2014.384 . 25517912 PMC4449828

[9] Inker LA, Astor BC, Fox CH, Isakova T, Lash JP, Peralta CA, et al KDOQI US commentary on the 2012 KDIGO clinical practice guideline for the evaluation and management of CKD. Am J Kidney Dis. 2014;63(5):713–35. Epub 2014/03/22. 10.1053/j.ajkd.2014.01.416 . 24647050

[10] Xia J, Wang L, Ma Z, Zhong L, Wang Y, Gao Y, et al Cigarette smoking and chronic kidney disease in the general population: a systematic review and meta-analysis of prospective cohort studies. Nephrol Dial Transplant. 2017;32(3):475–87. Epub 2017/03/25. 10.1093/ndt/gfw452 . 28339863

[11] Duncan MS, Freiberg MS, Greevy RA Jr., Kundu S, Vasan RS, Tindle HA. Association of Smoking Cessation With Subsequent Risk of Cardiovascular Disease. Jama. 2019;322(7):642–50. Epub 2019/08/21. 10.1001/jama.2019.10298 . 31429895 PMC6704757

[12] Kawachi I, Colditz GA, Stampfer MJ, Willett WC, Manson JE, Rosner B, et al Smoking cessation and time course of decreased risks of coronary heart disease in middle-aged women. Arch Intern Med. 1994;154(2):169–75. Epub 1994/01/24. . 8285812

[13] Rosenberg L, Kaufman DW, Helmrich SP, Shapiro S. The risk of myocardial infarction after quitting smoking in men under 55 years of age. N Engl J Med. 1985;313(24):1511–4. Epub 1985/12/12. 10.1056/NEJM198512123132404 . 4069159

[14] Rosenberg L, Palmer JR, Shapiro S. Decline in the risk of myocardial infarction among women who stop smoking. N Engl J Med. 1990;322(4):213–7. Epub 1990/01/25. 10.1056/NEJM199001253220401 . 2294448

[15] Kenfield SA, Stampfer MJ, Rosner BA, Colditz GA. Smoking and smoking cessation in relation to mortality in women. Jama. 2008;299(17):2037–47. Epub 2008/05/08. 10.1001/jama.299.17.2037 . 18460664 PMC2879642

[16] Nash SH, Liao LM, Harris TB, Freedman ND. Cigarette Smoking and Mortality in Adults Aged 70 Years and Older: Results From the NIH-AARP Cohort. Am J Prev Med. 2017;52(3):276–83. Epub 2016/12/05. 10.1016/j.amepre.2016.09.036 . 27914770 PMC5318256

[17] Haroun MK, Jaar BG, Hoffman SC, Comstock GW, Klag MJ, Coresh J. Risk factors for chronic kidney disease: a prospective study of 23,534 men and women in Washington County, Maryland. J Am Soc Nephrol. 2003;14(11):2934–41. Epub 2003/10/22. 10.1097/01.asn.0000095249.99803.85 . 14569104

[18] Fox CS, Larson MG, Leip EP, Culleton B, Wilson PW, Levy D. Predictors of new-onset kidney disease in a community-based population. Jama. 2004;291(7):844–50. Epub 2004/02/19. 10.1001/jama.291.7.844 . 14970063

[19] Hippisley-Cox J, Coupland C. Predicting the risk of chronic Kidney Disease in men and women in England and Wales: prospective derivation and external validation of the QKidney Scores. BMC Fam Pract. 2010;11:49 Epub 2010/06/23. 10.1186/1471-2296-11-49 . 20565929 PMC2905345

[20] Ryoo JH, Choi JM, Oh CM, Kim MG. The association between uric acid and chronic kidney disease in Korean men: a 4-year follow-up study. J Korean Med Sci. 2013;28(6):855–60. Epub 2013/06/19. 10.3346/jkms.2013.28.6.855 . 23772149 PMC3678001

[21] Tozawa M, Iseki K, Iseki C, Oshiro S, Ikemiya Y, Takishita S. Influence of smoking and obesity on the development of proteinuria. Kidney Int. 2002;62(3):956–62. Epub 2002/08/08. 10.1046/j.1523-1755.2002.00506.x . 12164878

[22] Regalado M, Yang S, Wesson DE. Cigarette smoking is associated with augmented progression of renal insufficiency in severe essential hypertension. Am J Kidney Dis. 2000;35(4):687–94. Epub 2000/03/31. 10.1016/s0272-6386(00)70017-5 . 10739791

[23] Briganti EM, Branley P, Chadban SJ, Shaw JE, McNeil JJ, Welborn TA, et al Smoking is associated with renal impairment and proteinuria in the normal population: the AusDiab kidney study. Australian Diabetes, Obesity and Lifestyle Study. Am J Kidney Dis. 2002;40(4):704–12. Epub 2002/09/27. 10.1053/ajkd.2002.35677 . 12324904

[24] Pinto-Sietsma SJ, Mulder J, Janssen WM, Hillege HL, de Zeeuw D, de Jong PE. Smoking is related to albuminuria and abnormal renal function in nondiabetic persons. Ann Intern Med. 2000;133(8):585–91. Epub 2000/10/18. 10.7326/0003-4819-133-8-200010170-00008 . 11033585

[25] Bleyer AJ, Shemanski LR, Burke GL, Hansen KJ, Appel RG. Tobacco, hypertension, and vascular disease: risk factors for renal functional decline in an older population. Kidney Int. 2000;57(5):2072–9. Epub 2000/05/03. 10.1046/j.1523-1755.2000.00056.x . 10792626

[26] Jin A, Koh WP, Chow KY, Yuan JM, Jafar TH. Smoking and risk of kidney failure in the Singapore Chinese health study. PLoS One. 2013;8(5):e62962 Epub 2013/05/15. 10.1371/journal.pone.0062962 . 23671645 PMC3650019

[27] Hallan SI, Orth SR. Smoking is a risk factor in the progression to kidney failure. Kidney Int. 2011;80(5):516–23. Epub 2011/06/17. 10.1038/ki.2011.157 . 21677635

[28] Nakanishi N, Fukui M, Tanaka M, Toda H, Imai S, Yamazaki M, et al Low urine pH Is a predictor of chronic kidney disease. Kidney Blood Press Res. 2012;35(2):77–81. Epub 2011/09/14. 10.1159/000330487 . 21912182

[29] Kim Y, Han BG. Cohort Profile: The Korean Genome and Epidemiology Study (KoGES) Consortium. Int J Epidemiol. 2017;46(2):e20 Epub 2016/04/17. 10.1093/ije/dyv316 . 27085081 PMC5837648

[30] Chandola T, Brunner E, Marmot M. Chronic stress at work and the metabolic syndrome: prospective study. Bmj. 2006;332(7540):521–5. Epub 2006/01/24. 10.1136/bmj.38693.435301.80 . 16428252 PMC1388129

[31] Shankar A, Klein R, Klein BE. The association among smoking, heavy drinking, and chronic kidney disease. Am J Epidemiol. 2006;164(3):263–71. Epub 2006/06/16. 10.1093/aje/kwj173 . 16775042

[32] Matsumoto A, Nagasawa Y, Yamamoto R, Shinzawa M, Hasuike Y, Kuragano T, et al The association of alcohol and smoking with CKD in a Japanese nationwide cross-sectional survey. Hypertens Res. 2017;40(8):771–8. Epub 2017/03/10. 10.1038/hr.2017.25 . 28275237

[33] Critchley J, Capewell S. Smoking cessation for the secondary prevention of coronary heart disease. Cochrane Database Syst Rev. 2004;(1):Cd003041 Epub 2004/02/20. 10.1002/14651858.CD003041.pub2 . 14974003

[34] Dawber TR. Summary of recent literature regarding cigarette smoking and coronary heart disease. Circulation. 1960;22:164–6. Epub 1960/07/01. . 13814553

[35] Doyle JT, Dawber TR, Kannel WB, Heslin AS, Kahn HA. Cigarette smoking and coronary heart disease. Combined experience of the Albany and Framingham studies. N Engl J Med. 1962;266:796–801. Epub 1962/04/19. 10.1056/nejm196204192661602 . 13887664

[36] Fenelon A, Preston SH. Estimating smoking-attributable mortality in the United States. Demography. 2012;49(3):797–818. Epub 2012/05/23. 10.1007/s13524-012-0108-x . 22610474 PMC3809994

[37] Tverdal A, Thelle D, Stensvold I, Leren P, Bjartveit K. Mortality in relation to smoking history: 13 years’ follow-up of 68,000 Norwegian men and women 35–49 years. J Clin Epidemiol. 1993;46(5):475–87. Epub 1993/05/01. 10.1016/0895-4356(93)90025-v . 8501474

[38] Statistics OfN. Adult smoking habits in the UK: 2018 2018. https://www.ons.gov.uk/peoplepopulationandcommunity/healthandsocialcare/healthandlifeexpectancies/bulletins/adultsmokinghabitsingreatbritain/2018.

[39] Prevention CfDCa. Current Cigarette Smoking Among Adults in the United States 2017. https://www.cdc.gov/tobacco/data_statistics/fact_sheets/adult_data/cig_smoking/index.htm.

[40] Ministry of Health and Welfare KNHaNES. Current cigarette smoking: ≥19 years, by sex 2019 [cited 2019]. http://kosis.kr/statHtml/statHtml.do?orgId=117&tblId=DT_11702_N001&vw_cd=MT_ETITLE&list_id=D1_3_1_1&scrId=&seqNo=&language=en&obj_var_id=&itm_id=&conn_path=A6&path=%252Feng%252Fsearch%252FsearchList.do.

