# Supplementary material for: Association of smoking with incident CKD risk in the general population: A community-based cohort study
Source: PLoS One. 2020 Aug 27;15(8):e0238111. doi: 10.1371/journal.pone.0238111 (PMC7451569; doi:10.1371/journal.pone.0238111)
Supplement: S1 Table — (DOCX) [file pone.0238111.s001.docx]

**S1 Table.** Type of missing data in main analysis data

| Variables | **Number of missing data** | | | |
| --- | --- | --- | --- | --- |
|  | Total | Never smokers | Ex-smokers | Current smokers |
| **Creatinine, n (%)** | 215 (2.5) | 117 (2.3) | 26 (1.9) | 72 (3.3) |
| **Proteinuria, n (%)** | 215 (2.5) | 131 (2.5) | 24 (1.8) | 60 (2.7) |
|  | | | | |
