# Supplementary material for: Association of smoking with incident CKD risk in the general population: A community-based cohort study
Source: PLoS One. 2020 Aug 27;15(8):e0238111. doi: 10.1371/journal.pone.0238111 (PMC7451569; doi:10.1371/journal.pone.0238111)
Supplement: S3 Table — (DOCX) [file pone.0238111.s003.docx]

**S3 Table.** Linear mixed model of annual eGFR decline according to time updated smoking status

|  | **Slope of eGFR decline (95% CI)** | ***p*-for-difference between groups** | | |
| --- | --- | --- | --- | --- |
|  |  | Never smokers | Ex-smokers | Current smokers |
| **Never smokers** | -1.345 (-1.405 to -1.286) | - |  |  |
| **Former smokers** | -1.616 (-1.681 to -1.551) | <0.001 | - |  |
| **Current smokers** | -1.731 (-1.764 to -1.698) | <0.001 | <0.001 | - |
| Slope of eGFR decline was presented as annual eGFR decline rate (mL/min/1.73m2/year).  Adjusted for age, sex, HTN, DM, alcohol, education, income, BMI, albumin, and total cholesterol.  BMI, albumin, and total cholesterol were treated as time varying covariates.  ***Abbreviations:*** eGFR, estimated glomerular filtration rate; HTN, hypertension; DM, diabetes mellitus; BMI, body mass index | | | | |
