# Supplementary material for: Association of smoking with incident CKD risk in the general population: A community-based cohort study
Source: PLoS One. 2020 Aug 27;15(8):e0238111. doi: 10.1371/journal.pone.0238111 (PMC7451569; doi:10.1371/journal.pone.0238111)
Supplement: S4 Table — (DOCX) [file pone.0238111.s004.docx]

**S4 Table.** Interaction terms of stratifying factors and the incident CKD risk of current smokers compared with never smokers

| **Subgroups** | | **N** | **Events (%)** | **HR (95% CI)** | ***p*-for-interaction** |
| --- | --- | --- | --- | --- | --- |
| Sex | Male | 2,841 | 525 (18.5) | 1.34 (0.99-1.83) | 0.062 |
|  | Female | 4,484 | 1,127 (25.1) |  |  |
| Age | Age≥60 years | 1,875 | 829 (44.2) | 1.08 (0.89-1.30) | 0.451 |
|  | Age<60 years | 5,450 | 823 (15.1) |  |  |
| Hypertnesion | Hypertensive | 1,040 | 386 (37.1) | 1.13 (0.90-1.40) | 0.297 |
|  | Non-hypertensive | 6,285 | 1,265 (20.1) |  |  |
| Daibetes | Diabetic | 442 | 194 (43.9) | 1.21 (0.92-1.60) | 0.177 |
|  | Non-diabetic | 6,883 | 1,457 (21.2) |  |  |
| SBP | SBP≥130mmHg | 2,105 | 683 (32.4) | 0.97 (0.80-1.18) | 0.767 |
|  | SBP<130mmHg | 5,220 | 969 (18.6) |  |  |
| BMI | BMI≥25kg/m^2^ | 3,164 | 797 (25.2) | 0.92 (0.76-1.11) | 0.360 |
|  | BMI<25kg/m^2^ | 4,161 | 855 (20.5) |  |  |
| Alcohol status | Current drinkers | 3,226 | 564 (17.5) | 0.98 (0.80-1.21) | 0.858 |
|  | Never+Ex-drinkers | 4,099 | 1,063 (25.9) |  |  |
| Income | Income≤3,000,000 won | 6,019 | 1,431 (23.8) | 1.14 (0.87-1.49) | 0.339 |
|  | Income>3,000,000 won | 1,306 | 221 (16.9) |  |  |
| Education | Education (low~middle) | 6,423 | 1,505 (23.4) | 0.89 (0.66-1.20) | 0.429 |
|  | Education (high) | 902 | 147 (16.3) |  |  |
| ***Abbreviations***: CKD, chronic kidney disease; HR, hazard ratio; CI, confidence interval; SBP, systolic blood pressure; BMI, body mass index | | | | | |
